# Single-copy detection of somatic variants from solid and liquid biopsy

**DOI:** 10.1038/s41598-021-85545-3

**Published:** 2021-03-16

**Authors:** Ana-Luisa Silva, Paulina Klaudyna Powalowska, Magdalena Stolarek, Eleanor Ruth Gray, Rebecca Natalie Palmer, Bram Herman, Cameron Alexander Frayling, Barnaby William Balmforth

**Affiliations:** 1 Biofidelity Ltd, 330 Cambridge Science Park, Cambridge, CB4 0WN UK; 2 Agilent Technologies LDA UK Ltd, 5500 Lakeside, Cheadle, SK8 3GR UK

**Keywords:** Cancer, Molecular biology

## Abstract

Accurate detection of somatic variants, against a background of wild-type molecules, is essential for clinical decision making in oncology. Existing approaches, such as allele-specific real-time PCR, are typically limited to a single target gene and lack sensitivity. Alternatively, next-generation sequencing methods suffer from slow turnaround time, high costs, and are complex to implement, typically limiting them to single-site use. Here, we report a method, which we term Allele-Specific PYrophosphorolysis Reaction (ASPYRE), for high sensitivity detection of panels of somatic variants. ASPYRE has a simple workflow and is compatible with standard molecular biology reagents and real-time PCR instruments. We show that ASPYRE has single molecule sensitivity and is tolerant of DNA extracted from plasma and formalin fixed paraffin embedded (FFPE) samples. We also demonstrate two multiplex panels, including one for detection of 47 *EGFR* variants. ASPYRE presents an effective and accessible method that simplifies highly sensitive and multiplexed detection of somatic variants.

## Introduction

Genotyping of somatic variants is standard of care for many cancer types, with results guiding the use of targeted and immune therapies. Test procedures commonly involve genetic analysis of either tumor tissue from a biopsy, or surgical resection, or circulating-tumor DNA (ctDNA) from a peripheral blood draw. Most commonly, tissue biopsies are used for initial genotyping because they directly sample the tumor. However, tissue biopsies also have limitations including associated morbidities and can be difficult to obtain, underestimate intratumor heterogeneity, have insufficient cell content, and DNA yields can be insufficient or low quality^1^. In contrast, analysis of plasma-derived ctDNA from a liquid biopsy is non-invasive and can more completely sample intratumor heterogeneity and metastases^2,3^ but has limited sensitivity if a patient has a low tumor burden or a low-shedding tumor. This makes analysis of early-stage tumors particularly challenging^4^, since, for many patients, current assays are insufficiently sensitive.

Tissue biopsies are subject to pathology review and those with insufficient tumor content, that cannot be rescued by macrodissection, are excluded from genetic testing. This ensures that clonal somatic variants in tested samples have variant allele fractions (VAFs) of at least 5–10%. In contrast, there is no equivalent quality control step for a peripheral blood draw, and clonal somatic variants exhibit a wide range of VAFs from < 0.1 to 10%.

Methods for somatic variant detection include real-time quantitative PCR (qPCR), digital PCR (dPCR) and next-generation sequencing (NGS). Real-time and dPCR are often limited to single genes. Allele-specific qPCR has a limit of detection around 1% and dPCR < 0.1% VAF^5–8^. Sequential testing of multiple single-gene assays can exhaust available material and increase costs, particularly if a repeat biopsy or blood draw is required^9–11^. Dividing material between multiple assays can also decrease assay sensitivity compared to using all available material in a single multiplexed assay. In this context, guidelines often support panel-based testing by NGS. Error-correction can decrease the limit of detection for NGS to < 0.1% VAF^12^. However, NGS workflows require significant investment in equipment, training and require complex bioinformatic analysis. In addition, turnaround times for NGS are around 13 days compared to 2–3 days for PCR assays^13^. In order to achieve clinically acceptable turnaround times, laboratories may batch less than the optimal number of samples in one sequencing run—in effect trading off cost against turnaround time.

To address these challenges, we developed Allele-Specific PYrophosphorolysis REaction (ASPYRE), a simple-to-use qualitative method for high-sensitivity detection across panels of somatic variants. The novel technology is based on pyrophosphorolysis, in which a high concentration of pyrophosphate ion is used to drive the DNA polymerisation reaction in reverse, resulting in the 3′–5′ de-polymerisation of double-stranded DNA^14^. This DNA digestion is extremely specific to perfectly matched double-stranded DNA, and is almost entirely inhibited by the presence of even a single mismatched base pair^15^, presenting an opportunity for highly specific detection of variants. The performance of the assay was tested against substitutions, insertions, deletions and gene fusions associated with non-small cell lung carcinoma (NSCLC) in both formalin-fixed paraffin-embedded (FFPE) tissue and contrived plasma samples. We also assessed the diagnostic sensitivity, specificity, and repeatability of the assay.

We report the successful demonstration of single-molecule detection of variants in plasma reference standards, as well as consistent performance of the technology between tissue and plasma across a wide range of DNA inputs. Overall, ASPYRE demonstrated sensitivity at, or in excess of, best in class molecular diagnostic assays including qPCR and NGS methods. In addition, ASPYRE supports higher multiplexing than qPCR assays used in routine practice, thereby avoiding the need to divide material between multiple assays. Compared to NGS, ASPYRE has low reagent costs, a simple laboratory and data analysis workflow, no requirement to batch multiple patient samples, and a rapid turnaround time. The full ASPYRE workflow takes four hours to complete, allowing timely return of results to physicians and patients.

## Results

### Allele-specific pyrophosphorolysis

**Figure 1:**
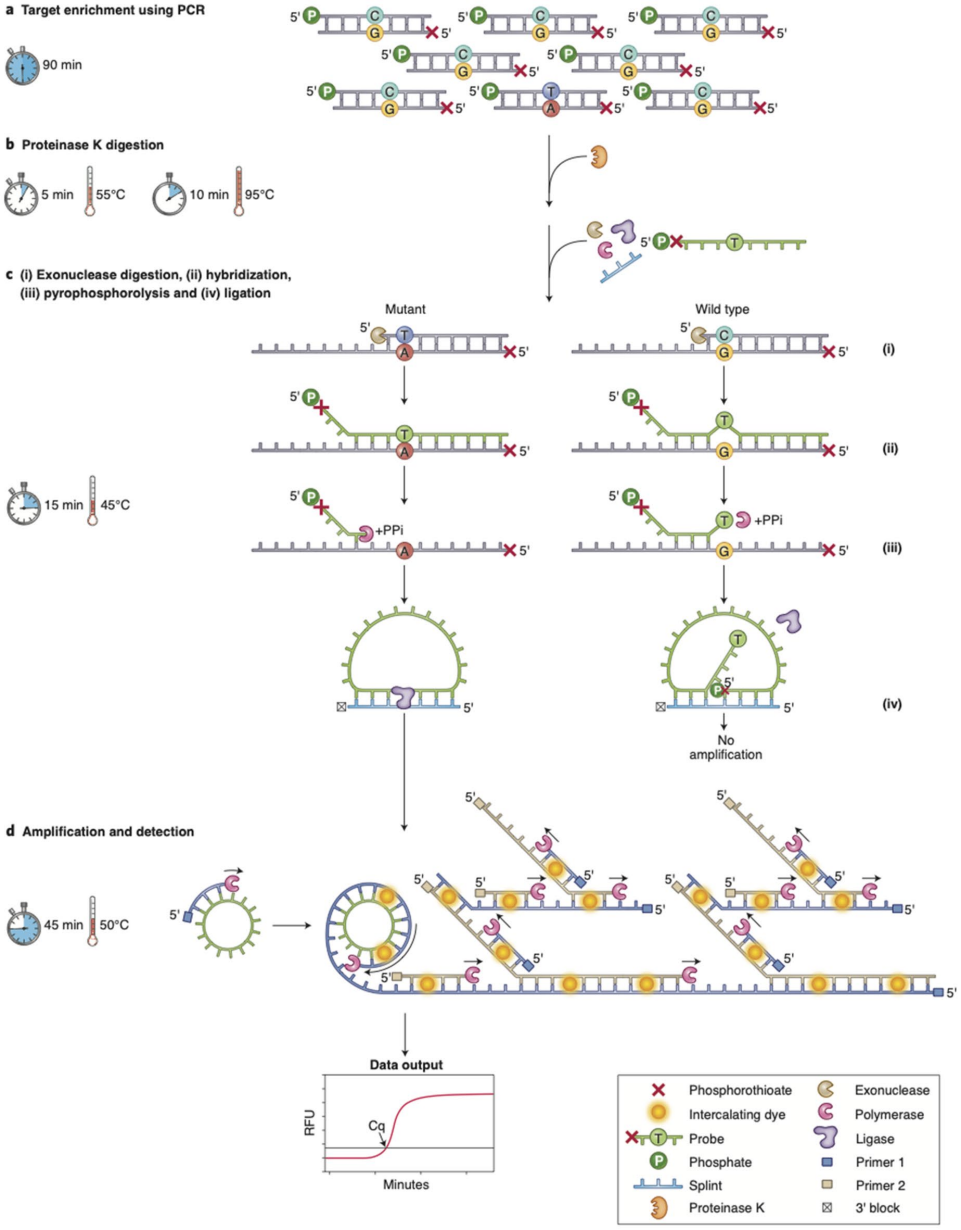
Overview of the ASPYRE assay. (**a**) Multiplex PCR amplification of wild-type and mutant alleles. (**b**) Enzymatic digestion of the PCR enzyme and subsequent heat-inactivation. (**c**) Exonuclease digestion to create single-stranded target molecules; hybridization of oligonucleotide probes to both mutant and wild-type sequences, with a mismatch to the wild-type sequence at the site of mutation; and pyrophosphorolysis of hybridized probes. (**d**) Circularization of only those probes that have been digested beyond the mutation site; and isothermal amplification and detection of circularized probes. Artwork by Debbie Maizels, Zoobotanica Scientific Illustration.

The ASPYRE assay consists of four reactions (Fig. 1). First, targets are amplified by PCR using primers that amplify both mutant and wild-type molecules (Fig. 1a). Second, remaining DNA polymerase is enzymatically digested (Fig. 1b). Third, PCR amplicons are made single-stranded by exonuclease digestion, probes that match the target variant are hybridized to the single-stranded DNA, and pyrophosphorolysis is performed using a DNA polymerase without exonuclease activity. Pyrophosphorolysis removes bases 5′ of the variant position only from probes that are perfectly matched to variant molecule(s). In contrast, pyrophosphorolysis stops at the mismatched position for probes that are imperfectly matched to wild-type molecule(s) (Fig. 1c). Fourth, perfectly matched probes that have been subject to pyrophosphorolysis are hybridized to a splint oligonucleotide and ligated to form circular single-stranded DNA that is isothermally amplified using universal priming sequences on each probe (Fig. 1d). The isothermal amplification is monitored in a standard real-time PCR instrument using a fluorescent intercalating dye. Using routine real-time PCR software and algorithms, a threshold and Cq values are defined^16^ and used to determine the presence or absence of the variant.

### Threshold determination

**Figure 2:**
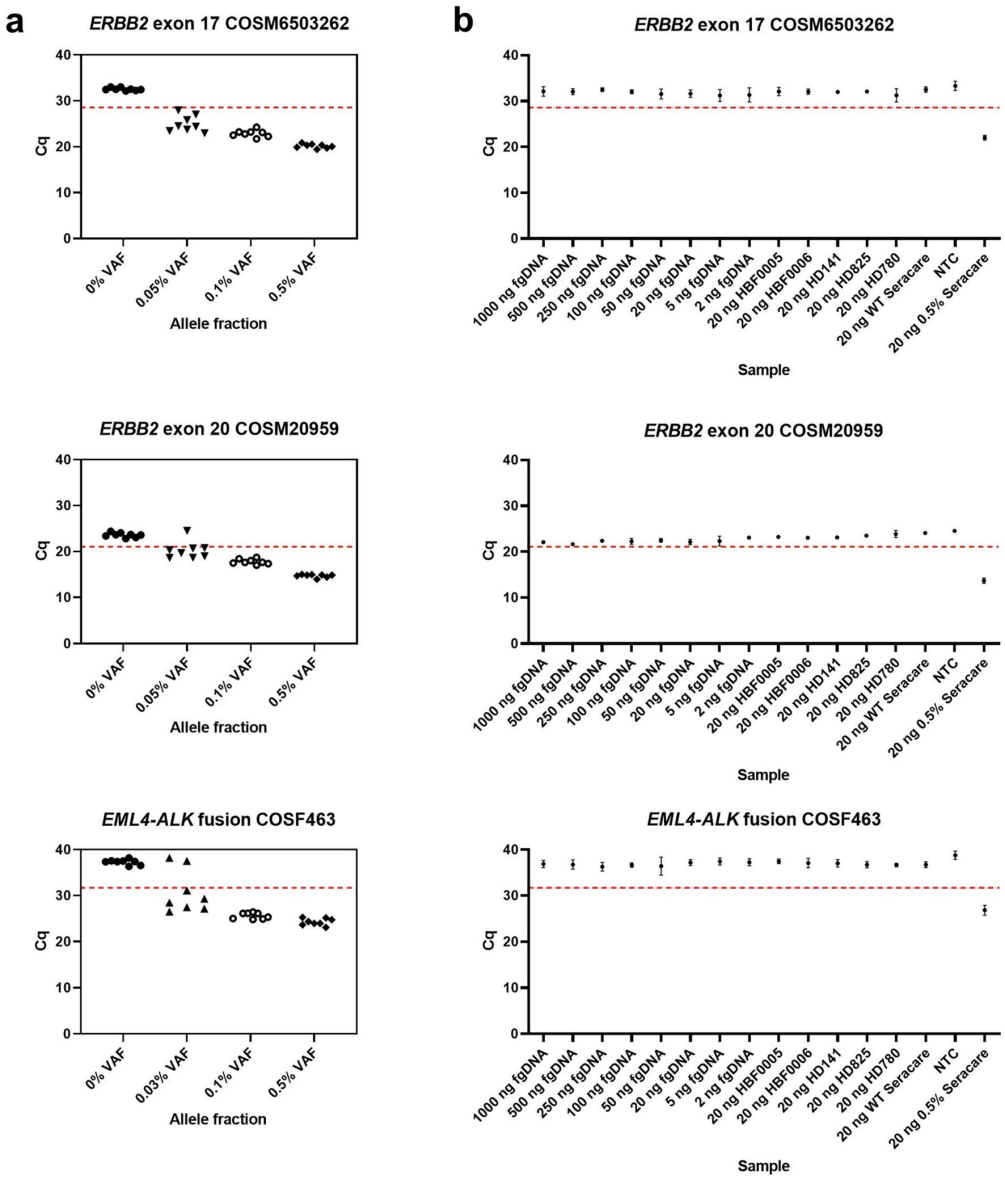
(**a**) Initial validation of ASPYRE for detection of *ERBB2* exons 17 and 20 mutations and *EML4-ALK* fusion in cfDNA-like samples. Each shape represents a different VAF, with each point representing one of the eight independent replicates performed. The red dashed line represents the threshold Cq used to determine the presence of variants. Results below the red dashed line indicate detection of the variant. (**b**) Mean Cq values and standard deviations for eight independent repeats of wild-type samples. Samples included human genomic DNA fragmented by ultrasonication (fgDNA) at varying DNA inputs and 20 ng of the following samples: FFPE extracted DNA (HBF0005, HBF0006); wild-type reference standard FFPE curl (HD141); cfDNA reference standards (HD825, HD780, WT Seracare). A positive control (0.5% Seracare) and no template control (NTC) were also analysed. The red dashed line represents the threshold Cq values defined in the initial validation work. Results below the red dashed line indicate detection of the variant.

Accurate detection of variant molecules requires a threshold that distinguishes reactions containing one, or more, variant molecules from reactions containing only wild-type molecules. To define assay thresholds, we used 20 ng of custom cell-free (cfDNA) reference standard that mimics post-extraction yield and fragment size distributions found in liquid biopsies (SeraCare Life Sciences). Three variants associated with NSCLC were amplified in a multiplex PCR; including one *ERBB2* exon 17 substitution, one *ERBB2* exon 20 insertion, and one *EML4*-*ALK* fusion. We assessed three VAFs (0%, 0.1% and 0.5%) using eight replicates of each (total 24 PCR reactions). Following the initial PCR, and enzymatic clean up, reactions were split into three tubes, each detecting one variant. For all variants, the resulting quantification cycle (Cq) values from reactions containing only wild-type template (0% VAF) were clearly separable from those including variant molecules (0.1% and 0.5% VAF) (Fig. 2a). We set Cq thresholds for each of the variants at the lower value of either five standard deviations below the mean of the wild-type reaction or two standard deviations above the mean of the 0.1% VAF samples.

We next assessed the selected thresholds and assay background using a range of sample types and DNA inputs. We assessed DNA inputs from 2 ng to 1 µg using wild-type human genomic DNA ultrasonicated to ~ 150 bp. Additional samples included FFPE from patients with NSCLC, wild-type reference standard FFPE curl material, cfDNA reference standards, in addition to positive (0.5% VAF) and no template control reactions. Eight independent repeats were used for each reaction (total 112 PCR reactions). Consistent with previous results, all three variants were detected in all replicates of the 0.5% VAF cfDNA reference standard (Fig. 2b). None of the variants were detected in any of the wild-type sample replicates, giving 100% specificity. Importantly, the Cq values for each of the variants were consistent across all replicates, DNA inputs and sample types, including FFPE and cfDNA-like material.

### Accurate detection of variants from single molecules

We next sought to determine whether ASPYRE could detect single variant molecules against a background of wild-type DNA. We used the three NSCLC variants, described above, and reduced custom cfDNA reference standard DNA input to 2 and 5 ng. Substitutions and insertions were assessed at 0%, 0.07%, 0.1% and 0.5% VAF and the fusion at 0%, 0.05%, 0.1% and 0.5% VAF. Each condition was assessed across 48 replicates by two operators using different reagent lots over 2 days (total 768 PCR reactions). The reference input and VAFs were chosen such that only a proportion of PCR reactions included one, or more, variant molecule, with the majority predicted to contain either zero or one variant molecule.

We applied the previously determined threshold Cq values for each variant to experimental results and counted the number of positive and negative reactions. The number of positive results from different operators and reagent lots were within statistical noise of equal (ANOVA F-test, *P* = 0.5). All wild-type samples (0% VAF) presented as negative, giving a specificity of 100%. For the remaining samples, we estimated the mean number of intact variant molecules that could be amplified by PCR in each reaction (“Methods”). We then used probit regression to estimate the 95% limit of detection. LoD95 results were 5.2 variant molecules for *ERBB2* exon 17, 4.0 for *ERBB2* exon 20, and 2.9 for the *EML4*-*ALK* fusion. We then compared our results to an ideal assay that perfectly detects variant molecules that can be amplified by PCR, failing only when there are no such molecules in the sample (“Methods”). In this case, sensitivity is dependent only on sampling and the LoD95 is 3.0 variant molecules. These results indicate that ASPYRE was able to detect molecules with single molecule sensitivity. In further support of this hypothesis, for all three combinations of two variants, the probability that the same sample was positive for both variants was consistent with independent sampling of variant molecules (Cochran–Mantel–Haenszel test: *ERBB2* exon 17 and *ERBB2* exon 20 *P* = 0.5; *ERBB2* exon 17 and *EML4*-*ALK P* = 0.4; *ERBB2* exon 20 and *EML4*-*ALK P* = 0.5). The exception was one sample at 0.5% VAF with 5 ng input, which was negative for all three variants. This result is likely an assay failure, given the number of input variant molecules, and allows us to estimate a false-negative rate of 1%. In future, use of a positive control will allow identification of assay failures. Taken together, these data indicate that ASPYRE can achieve detection of a single molecule of variant DNA against a wild-type background, with high sensitivity and specificity.

### *EGFR* panel

We next applied ASPYRE to detection of 47 *EGFR* variants, commonly used in treatment selection for NSCLC. These included 46 deletions in exon 19 and the L858R (COSM6224) mutation in exon 21^17^. Two target regions of *EGFR* were co-amplified by PCR and reactions were subject to enzymatic clean up. Each reaction was then split into 47 tubes, one detecting L858R and the other 46 each detecting a single deletion.

**Figure 3:**
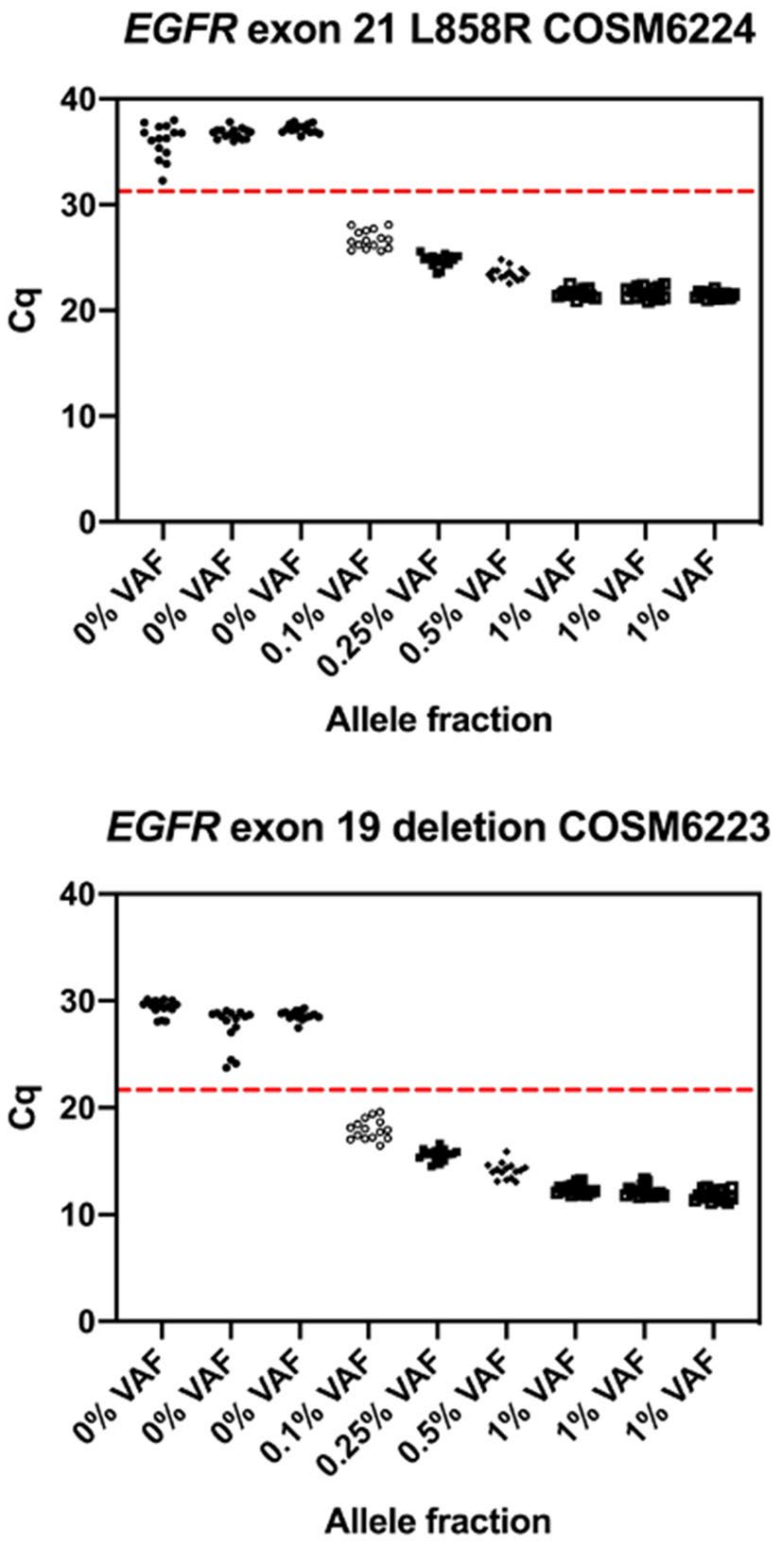
Cq values for (**a**) EGFR exon 21 L858R (COSM6224) and (**b**) exon 19 (COSM6223) deletion. The red dashed line represents the threshold Cq used to determine the presence of variants. Results below the red dashed line indicate detection of the variant.

We performed six DNA extractions on FFPE treated cell-line mixtures, including three at 0% VAF (HD141) and three at 1% VAF (HD850). No sample-to-sample variation was observed between the independent DNA extractions for FFPE cell-lines (Supplementary Fig. 1). We mixed the cell-lines to give 0.5%, 0.25% and 0.1% VAF samples. Each of the six DNA extractions, and three mixes, were PCR amplified in 5 replicates including 20 ng DNA input (total 45 PCR reactions). Each PCR reaction was then used in 3 replicate detection reactions (total 135 detection reactions). Cq thresholds for each of the variants were set at five standard deviations below the mean of the 0% VAF replicates. Results demonstrated 100% sensitivity at all VAFs including 0.1% (Fig. 3).

We next tested the performance of ASPYRE on patient samples. We analyzed six NSCLC FFPE tissue samples, with known variants, sourced from a commercial biobank; one positive control *EGFR* reference standard (HD850); and one negative control (DNA fragmented by ultrasonication, fgDNA). Patient DNA samples were found to be more fragmented than FFPE treated cell-line mixtures (Supplementary Fig. 1). We PCR amplified 20 ng input DNA from each sample and control using six replicates (total 48 PCR reactions). We first analyzed amplification products using polyacrylamide gel electrophoresis (PAGE) (Supplementary Fig. 2). No deletion product was observed for the positive control HD850, which had an *EGFR* exon 19 deletion with a VAF of 1%. Deletion products were visible for patient samples HBF003 and HBF005, consistent with high VAF *EGFR* exon 19 deletions. We next analyzed results from ASPYRE, using Cq thresholds for each of the variants that were set at five standard deviations below the mean of the negative control. Despite not being visible by PAGE, the positive control 1% EGFR exon 19 deletion was detected by ASPYRE. Further analysis of the patient samples identified three false positives, including two L858R replicates from HBF0004 and one COSM12413 replicate from HBF0001 (Supplementary Fig. 3). Despite this, Cq values for the false positive L858R replicates were visually separable from the positive control samples, suggesting that our threshold Cq values could be improved. For patient HBF0006, we also failed to detect an exon 19 deletion, both on PAGE and in all six replicates using ASPYRE, that was previously identified by a biobank using qPCR. To investigate this discrepancy, the biobank repeated qPCR analysis on their original DNA extraction, used for sample characterization, and our subsequent DNA extraction used for ASPYRE. The exon 19 deletion was weakly detected in the biobank’s original DNA extraction but not in our ASPYRE DNA extraction. This is consistent with sample contamination, a well-known issue when handling FFPE blocks^18^. Overall, sensitivity was 100%, specificity was 99.8% and concordance was 99.8%. Taken together, these results illustrate that ASPYRE is capable of detecting clinically relevant *EGFR* variants in FFPE and contrived cfDNA samples with high sensitivity, specificity and concordance.

## Discussion

In this study, we have demonstrated that the ASPYRE technology has high sensitivity and specificity for the detection of SNVs, indels and gene fusions, coupled with a wide tolerance to the sample type, DNA fragmentation profile, and DNA input quantity.

Threshold Cq values defined in the validation study were successfully applied to all DNA sample types and DNA inputs. A consistent background signal was obtained for assays with differing sample types, differing DNA inputs and in the absence of template. This stands in contrast to PCR-based assays, which often suffer from carryover of inhibitors from FFPE samples^19^ and have background and signal Cq values that depend on input DNA concentration. The background signal of ASPYRE is consistent with non-specific amplification of the probe during the isothermal detection step^20^. Further optimization of reaction conditions, including use of molecular beacon probes, rather than an intercalating dye^21^, could reduce background fluorescence. Using 20 ng of input DNA, ASPYRE had 100% sensitivity and 100% specificity for the tested substitution, insertion and fusion variants at 0.5% and 0.1% VAF. We further investigated assay sensitivity by limiting dilution and used probit regression to demonstrate that the LoD95 was similar to that expected from Poisson sampling. This indicates that ASPYRE is capable of detecting single copies of variant molecules. Sensitivity and specificity values are significantly higher than those achieved by real-time PCR and match, or exceed, dPCR and NGS assays. This offers the potential to detect variants at low fractions in cfDNA (for patient stratification, monitoring and early detection) or tissue samples with low cellularity such as fine needle aspirates.

We also tested NSCLC FFPE patient samples for *EGFR* mutations. Specificity was 99.8% but could be further improved by defining thresholds using negative control FFPE DNA samples rather than ultrasonicated DNA, which might have poor commutability^22^. One sample was not concordant between ASPYRE and previous qPCR results. Re-analysis of DNA extractions using qPCR confirmed the ASPYRE finding, providing 100% sensitivity and 100% concordance. Amongst our studies, we found one sample that was unexpectedly negative for all variants. This allowed us to estimate a false-negative rate of 1%. In future, including a positive control in the assay will allow straightforward identification of such assay failures.

Several assays in standard clinical usage are based on Amplification Refractory Mutation System (ARMS)^23^. These include Roche cobas and Qiagen therascreen single gene assays, with reported analytical LoD95 of > 3% for detection of *EGFR* substitutions and indels (FDA Summary of Safety and Effectiveness Data cobas EGFR Mutation Test v2 and therascreen EGFR RGQ PCR Kit). In addition, enhanced allele-specific qPCR approaches have been described with improved sensitivity compared to ARMS. For example, competitive allele-specific TaqMan PCR has a reported LoD of 0.1% for *EGFR* L858R and 1% for T790M^24^. Similarly, modified blocking oligonucleotides have been applied to reduce the LoD to 0.1% for four common *KRAS* variants^25^. Compared to ASPYRE, these modified qPCR approaches have limited multiplexing. In an alternative approach, Blocker Displacement Amplification (BDA)^26^ has a reported LoD of 0.1% and could potentially support multiplexing of multiple genes. However, BDA multiplexing is either limited by the number of independent colour channels on a qPCR instrument or requires an alternative readout, such as NGS, thereby increasing turnaround time and assay complexity.

A comparison of ASPYRE to existing assays is shown in Table 1. ASPYRE is simple, low-cost, can be implemented on real-time PCR instrumentation, and is ideally suited to multi-gene panels of clinically actionable variants. The method supports higher multiplexing than either allele-specific qPCR or dPCR, thereby avoiding dividing material between multiple assays with the associated decrease in sensitivity and increased assay costs. Our current implementation still has limitations, compared to NGS, in the number of targets that can be multiplexed and the requirement for prior knowledge of variants. Multiplexing can be addressed by parsing PCR reactions into smaller microfluidic detection reactions, although this level of multiplexing is not essential for all tumor types. For example, NCCN guidelines for NSCLC recommend testing for substitutions in *EGFR*, *BRAF* and *KRAS*; gene fusions in *ALK*, *RET*, *ROS1*, and *NTRK1-3*; and *MET* exon 14 skipping^17^. In this context, broader panels might not provide additional benefit, with identification of somatic variants with no known drug association having limited clinical significance. A further limitation is that we have not assessed copy number amplification: high level amplification of *MET* is an emerging biomarker for NSCLC^17^. Compared to NGS, ASPYRE has a simple laboratory workflow, requiring straightforward sequential addition of reagents without intermediate purification steps, and does not require complex bioinformatic interpretation. The assay can be run in less than 4 h and is sufficiently cost effective to run a wide range of batch sizes from one to hundreds of samples. We anticipate that ASPYRE will help address clinical needs for highly accurate multiplexed detection of somatic variants with reductions in assay complexity allowing laboratories of all sizes to rapidly test patients.

**Table 1:** Comparison of most frequently used analysis methods with ASPYRE assay.

	Targeted NGS	Allele-specific qPCR	dPCR	ASPYRE
Sensitivity (%)	< 0.1	~ 1	< 0.1	< 0.1
Multiplexing	1000+	< 20	< 6	50+
Requires prior knowledge of variant	No	Yes	Yes	Yes
Instrument complexity	High	Low	Medium	Low
Laboratory workflow complexity	High	Low	Medium	Medium
Data analysis complexity	High	Low	Low	Low
Time to result	1–5 days	2 h	2 h	4 h
Cost	High	Low	Medium	Low

## Methods

### Samples

Custom cell-free DNA reference standards were supplied by SeraCare Life Sciences. Standards included either 0%, 0.1% and 0.5% VAF for *ERBB2* substitution (COSM6503262), *ERBB2* insertion (COSM20959), and *EML4*-*ALK* fusion (COSF463). COSM and COSF are COSMIC identifiers^27^. Mean fragment sizes were 170 bp for the 0% VAF sample, 157 bp for the 0.1% VAF sample, and 155 bp for the 0.5% VAF sample. Samples with VAFs lower than 0.1% were prepared by dilution of the 0.1% VAF sample with the 0% VAF (wild-type) sample. The 0.1% VAF sample had two additional copies of *ERBB2* compared to the *EML4*-*ALK* fusion, therefore diluted samples had VAFs of 0.07% for *ERBB2* substitutions and insertions and 0.05% for *EML4*-*ALK* fusions.

Human genomic DNA was supplied by Promega (G3041) and fragmented by ultrasonication to a mean size of ~ 150 bp to mimic post-extraction size distribution from a liquid biopsy sample. FFPE cell-lines were supplied by Horizon Discovery and included 100% *EGFR* wild type FFPE DNA (HD141) and 1% *EGFR* Quantitative Multiplex FFPE Reference Standard (HD850). Six FFPE blocks from patients with confirmed NSCLC adenocarcinoma were supplied by BioIVT (Supplementary Table 1). Cell-free cfDNA reference standards included EGFR Multiplex cfDNA Reference Standard Set (HD825) and Multiplex I cfDNA Reference Standard Set (HD780).

### DNA extraction and quantitation

The ReliaPrep FFPE gDNA Miniprep System (Promega) was used to extract DNA from both FFPE cell-lines and FFPE tissue samples. DNA was quantitated using a fluorescent dye specific to double-stranded DNA (Qubit dsDNA HS assay, Thermo Fisher Scientific) and assessed by Genomic DNA ScreenTape Assay on a 4200 TapeStation (Agilent).

### Initial target amplification and proteinase K treatment

PCR primers were designed to minimise amplicon lengths, in order to increase the probability of amplifying target molecules in fragmented DNA. We designed 10 forward and reverse primer sequences, where the primer 3′ end was positioned 20–30 nucleotides from the variant, and empirically tested all possible combinations.

PCR reactions were prepared by mixing 10 µL HF buffer (F555, Thermo Fisher Scientific), 0.5 µL MgSO_4_ (100 mM, B1003, New England Biolabs), 2 µL primers (10 µM each), 2 µL dNTPs mix (10 mM, Promega), 0.5 µL Phusion U Hot Start Polymerase (F555, Thermo Fisher Scientific), 0.5 µL Antarctic Thermolabile UDG (1 U/µL, M0372, New England Biolabs) and 2–20 ng DNA template in a 50 µL reaction.

PCR amplification of *EGFR* exons 19 and 20 included primers 5′-A*A*G*TTAAAATTCCCGTCGCTA-3′, 5′-/P/AGCAAAGCAGAAACTCACATCG-3′, 5′-A*C*A*CCGCAGCATGTCAAGAT-3′, and 5′-/P/GCCTCCTTCTGCATGGTATTCT-3′. Phosphorothioate bonds are denoted by an asterisk and phosphate groups by /P/. Cycling conditions (including UDG treatment) were 37 °C 10 min; 98 °C 1 min; 50 cycles of 98 °C 10 s, 57.5 °C 20 s, 72 °C 20 s; and 72 °C 5 min.

PCR amplification of ERBB2 exons 17 and 20, and EML4-ALK included primers 5′-C*C*A*AAGACCACCCCCAAGAC-3′, 5′-/P/GCCCTCTGACGTCCATCATC-3′, 5′-C*C*C*CAGGAAGCATACGTGAT-3′, 5′-/P/GAAGGCGGGAGACATATGGG-3′, 5′-T*C*A*TTTCTGGTAATTCTCACAT-3′ and 5′-/P/CTGTAGGCAGGGATGGTAACTCCT-3′. Cycling conditions (including UDG treatment) were 37 °C 10 min; 98 °C 1 min; 50 cycles of 98 °C 10 s, 57 °C 15 s, 72 °C 15 s; and 72 °C 5 min.

After PCR, 40 µL of PCR mix was added to 50 µL of proteinase K mix in a total volume of 90 µL. Proteinase K mix included 8 µL 5x A7 buffer (50 mM Tris–acetate pH 8.0, 125 mM potassium acetate, 25 mM magnesium acetate, 0.5% Triton X-100), 2.25 µL Proteinase K (P8107S, New England Biolabs) and 39.75 µL nuclease free water. Reactions were incubated at 55 °C for 5 min followed by heat inactivation at 95 °C for 10 min.

### Pyrophosphorolysis reaction

7.5 µL pyrophosphorolysis (PPL) mix was prepared by mixing 0.5 µL of 20 × BFF buffer (200 mM tris–acetate pH 7.0, 600 mM potassium acetate, 342.5 mM magnesium acetate, 2% Triton X-100), 0.25 µL sodium pyrophosphate dibasic (10 mM stock, Sigma-Aldrich), 0.02 µL Klenow Exo- (M0212, New England Biolabs), 0.03 µL Apyrase (M0398, New England Biolabs), 0.2 µL probe (1 µM stock), 0.3 µL ligation splint (1 µM stock), 0.1 µL E. coli Ligase (M0205, New England Biolabs), 0.2 µL Lambda exonuclease (M0262, New England Biolabs) and 5.9 µL nuclease free water. For *ERBB2* exon 20, the 7.5 µL PPL mix was adjusted to include 0.1 µL sodium pyrophosphate dibasic, 0.06 µL Klenow exo-, and 6.01 µL nuclease free water, in place of standard volumes. 2.5 µL of Proteinase K treated PCR sample was added to PPL mix at 4 °C to give a total volume of 10 µL. Reactions were incubated at 45 °C for 15 min before storage at 4ºC.

Probes included a constant 53 nucleotide 5′ sequence 5′-/P/A*T*G*TTCGATGAGCTTTGACAATACTTGATCGATGCAGATATAGGATGTTGCGA-3′ for isothermal amplification, followed by 18–20 nucleotides specific to the target sequence. Probes were designed to perfectly match mutant variants, while displaying at least one mismatch to wild-type molecules. Mismatches were positioned after empirically testing a number of probe and splint combinations, with final mismatches located from 2 to 25 bases from the 3′ of the probe. Splint oligonucleotides included a constant 20 nucleotide sequence 5′-TGTCAAAGCTCATCGAACAT-3′, where the 3′ base is mismatched to prevent pyrophosphorolysis, and the remaining 19 nucleotides are complementary to the 5′ of the probe sequence. The constant sequence is followed by 6–14 nucleotides complementary to the 3′ end of the probe oligonucleotide.

### Real-time isothermal amplification

10 µL of detection mixture was prepared by mixing 3 µL Thermopol buffer (B9004, New England Biolabs), 0.4 µL BST 2.0 WarmStart (M0538, New England Biolabs), 1.2 µL dNTPs mix (10 mM stock, Promega), 1.13 µL Syto82 dye (S11363, Thermo Fisher Scientific), 0.08 µL Thermostable inorganic pyrophosphatase (M0296, New England Biolabs), 0.16 µL of primer mix (20 µM stock, 5′-T*C*GCAACATCCTATATCTGC-3′ and 5′-T*G*AGCTTTGACAATACTTGA-3′) and 4.03 µL nuclease free water. 1.25 µL PPL reaction sample was added to 10 µL of detection mix. Samples were incubated at 50 °C for 60 min in a CFX384 Touch Real-Time PCR Detection System (Bio-Rad). The fluorescence read-out was taken every minute in the Cal Orange 560 setting.

### Data analysis

Quantification cycle (Cq) values were identified using the CFX Maestro software (Bio-Rad). Fluorescence data and Cq values were exported from CFX Maestro software into MS Excel for subsequent analysis. Data were plotted using Prism8 software. Statistical analyses were performed using Python statsmodels and scipy.

We estimated the mean number µ of variant molecules that can be amplified by PCR in each reaction using: µ = (number of haploid genomes) × (target copy number/2) × VAF × (intact fraction of input molecules). The number of haploid genomes was estimated from the input DNA mass, assuming that 3.3 pg is one haploid genome. The target copy number was 2 for diploid targets but can be altered by copy number variation. The intact fraction of input molecules, which include both PCR primer sites, was estimated using the length of the target amplicon, and the fragmentation profile of the input DNA. We assumed that fragmentation breakpoints were uniformly distributed. We therefore estimated the fraction of intact fragments using max(0, 1 − amplicon length/fragment length) averaged over the fragment length distribution. The estimated fraction of input molecules that were intact was 0.54 for *EML4*-*ALK*, 0.61 for *ERBB2* exon 17 and 0.69 for *ERBB2* exon 20.

To estimate the limit of detection, we performed probit regression on log(µ), as recommended by BloodPAC^28^. We also compare our assay to an ideal assay that always generates a positive signal in presence of one, or more, amplifiable variant molecule(s). Under this condition, the assay sensitivity is dependent on Poisson sampling and can be estimated using the mean number of variant molecules that can be amplified by PCR, giving sensitivity = 1 − exp(− µ).

## Supplementary Information

Supplementary Information.

## References

[1] Aggarwal C, Rolfo CD, Oxnard GR, Gray JE, Sholl LM, Gandara DR (2021). Strategies for the successful implementation of plasma-based NSCLC genotyping in clinical practice. Nat. Rev. Clin. Oncol..

[2] Gerlinger M, Rowan AJ, Horswell S, Math M, Larkin J, Endesfelder D (2012). Intratumor heterogeneity and branched evolution revealed by multiregion sequencing. N. Engl. J. Med..

[3] De Mattos-Arruda L, Weigelt B, Cortes J, Won HH, Ng CKY, Nuciforo P (2014). Capturing intra-tumor genetic heterogeneity by de novo mutation profiling of circulating cell-free tumor DNA: A proof-of-principle. Ann. Oncol..

[4] Bettegowda C, Sausen M, Leary RJ, Kinde I, Wang Y, Agrawal N (2014). Detection of circulating tumor DNA in early- and late-stage human malignancies. Sci. Transl. Med..

[5] Lang AH, Drexel H, Geller-Rhomberg S, Stark N, Winder T, Geiger K (2011). Optimized allele-specific real-time PCR assays for the detection of common mutations in KRAS and BRAF. J. Mol. Diagn..

[6] Watanabe M, Kawaguchi T, Isa S-I, Ando M, Tamiya A, Kubo A (2015). Ultra-sensitive detection of the pretreatment EGFR T790M mutation in non-small cell lung cancer patients with an EGFR-activating mutation using droplet digital PCR. Clin. Cancer Res..

[7] Wood-Bouwens C, Lau BT, Handy CM, Lee H, Ji HP (2017). Single-color digital PCR provides high-performance detection of cancer mutations from circulating DNA. J. Mol. Diagn..

[8] Seekhuntod S, Thavarungkul P, Chaichanawongsaroj N (2016). Validation of a multiplex allele-specific polymerase chain reaction assay for detection of KRAS gene mutations in formalin-fixed, paraffin-embedded tissues from colorectal cancer patients. PLoS ONE.

[9] Lindeman NI, Cagle PT, Aisner DL, Arcila ME, Beasley MB, Bernicker EH (2018). Updated molecular testing guideline for the selection of Lung Cancer patients for treatment with targeted tyrosine kinase inhibitors: Guideline from the College of American Pathologists, the International Association for the Study of Lung Cancer, and the Association for Molecular Pathology. Arch. Pathol. Lab. Med..

[10] Pennell NA, Mutebi A, Zhou Z-Y, Ricculli ML, Tang W, Wang H (2019). Economic impact of next-generation sequencing versus single-gene testing to detect genomic alterations in metastatic non-small-cell lung cancer using a decision analytic model. JCO Precis Oncol..

[11] Kalemkerian GP, Narula N, Kennedy EB, Biermann WA, Donington J, Leighl NB (2018). Molecular testing guideline for the selection of patients with Lung Cancer for treatment with targeted tyrosine kinase inhibitors: American society of clinical oncology endorsement of the College of American Pathologists/International Association for the Study of Lung Cancer/Association for Molecular Pathology clinical practice guideline update. J. Clin. Oncol..

[12] Newman AM, Bratman SV, To J, Wynne JF, Eclov NCW, Modlin LA (2014). An ultrasensitive method for quantitating circulating tumor DNA with broad patient coverage. Nat. Med..

[13] Rolfo C, Mack PC, Scagliotti GV, Baas P, Barlesi F, Bivona TG (2018). Liquid biopsy for advanced non-small cell lung cancer (NSCLC): A statement paper from the IASLC. J. Thorac. Oncol..

[14] Liu Q, Sommer SS (2004). Pyrophosphorolysis by Type II DNA polymerases: Implications for pyrophosphorolysis-activated polymerization. Anal. Biochem..

[15] Balmforth, B. Single nucleotide detection method. World Patent. /2020/016590 (2020).

[16] Bustin SA, Benes V, Garson JA, Hellemans J, Huggett J, Kubista M (2009). The MIQE guidelines: Minimum information for publication of quantitative real-time PCR experiments. Clin. Chem..

[17] Ettinger DS, Wood DE, Aggarwal C, Aisner DL, Akerley W, Bauman JR (2019). NCCN guidelines insights: Non-small cell lung cancer, version 1.2020. J. Natl. Compr. Cancer Netw..

[18] Vos HI, van der Straaten T, Coenen MJH, Flucke U, te Loo DMWM, Guchelaar H-J (2015). High-quality genotyping data from formalin-fixed, paraffin-embedded tissue on the drug metabolizing enzymes and transporters plus array. J. Mol. Diagn..

[19] Okello JBA, Zurek J, Devault AM, Kuch M, Okwi AL, Sewankambo NK (2010). Comparison of methods in the recovery of nucleic acids from archival formalin-fixed paraffin-embedded autopsy tissues. Anal. Biochem..

[20] Wang G, Ding X, Hu J, Wu W, Sun J, Mu Y (2017). Unusual isothermal multimerization and amplification by the strand-displacing DNA polymerases with reverse transcription activities. Sci. Rep..

[21] Nilsson M, Gullberg M, Dahl F, Szuhai K, Raap AK (2002). Real-time monitoring of rolling-circle amplification using a modified molecular beacon design. Nucleic Acids Res..

[22] Hardwick SA, Deveson IW, Mercer TR (2017). Reference standards for next-generation sequencing. Nat. Rev. Genet..

[23] Newton CR, Graham A, Heptinstall LE, Powell SJ, Summers C, Kalsheker N (1989). Analysis of any point mutation in DNA. The amplification refractory mutation system (ARMS). Nucleic Acids Res..

[24] Yang Y, Meng Y, Zhang H, Shen X, Li R, Yu L (2018). Detection of EGFR and BRAF mutations by competitive allele-specific TaqMan polymerase chain reaction in lung adenocarcinoma. Oncol. Lett..

[25] Chubarov AS, Oscorbin IP, Filipenko ML, Lomzov AA, Pyshnyi DV (2020). Allele-specific PCR for KRAS mutation detection using phosphoryl guanidine modified primers. Diagnostics (Basel)..

[26] Wu LR, Chen SX, Wu Y, Patel AA, Zhang DY (2017). Multiplexed enrichment of rare DNA variants via sequence-selective and temperature-robust amplification. Nat. Biomed. Eng..

[27] Tate JG, Bamford S, Jubb HC, Sondka Z, Beare DM, Bindal N (2019). COSMIC: The catalogue of somatic mutations in cancer. Nucleic Acids Res..

[28] Godsey JH, Silvestro A, Barrett JC, Bramlett K, Chudova D, Deras I (2020). Generic protocols for the analytical validation of Next-Generation Sequencing-based ctDNA assays: A joint consensus recommendation of the BloodPAC’s Analytical Variables Working Group. Clin. Chem..

