# Supplementary material for: Single-copy detection of somatic variants from solid and liquid biopsy
Source: Sci Rep. 2021 Mar 16;11:6068. doi: 10.1038/s41598-021-85545-3 (PMC7966381; doi:10.1038/s41598-021-85545-3)
Supplement: Supplementary file 1 — Supplementary Information. [file 41598_2021_85545_MOESM1_ESM.docx]

**[Supplementary Information]**

**Single-Copy Detection of Somatic Variants from Solid and Liquid Biopsy**

Ana-Luisa Silva^1^, Paulina Klaudyna Powalowska^1^, Magdalena Stolarek^1^, Eleanor Ruth Gray^1^, Rebecca Natalie Palmer^1^, Bram Herman^2^, Cameron Alexander Frayling^1^, Barnaby William Balmforth^1*^

^1^Biofidelity Ltd, Cambridge, United Kingdom

^2^Diagnostics and Genomics Group, Agilent Technologies Inc., Santa Clara, CA

^*^Corresponding author

Barnaby Balmforth, Biofidelity Ltd, 330 Cambridge Science Park, Cambridge, CB4 0WN, UK, +441223 981750, [b.balmforth@biofidelity.com](mailto:b.balmforth@biofidelity.com)


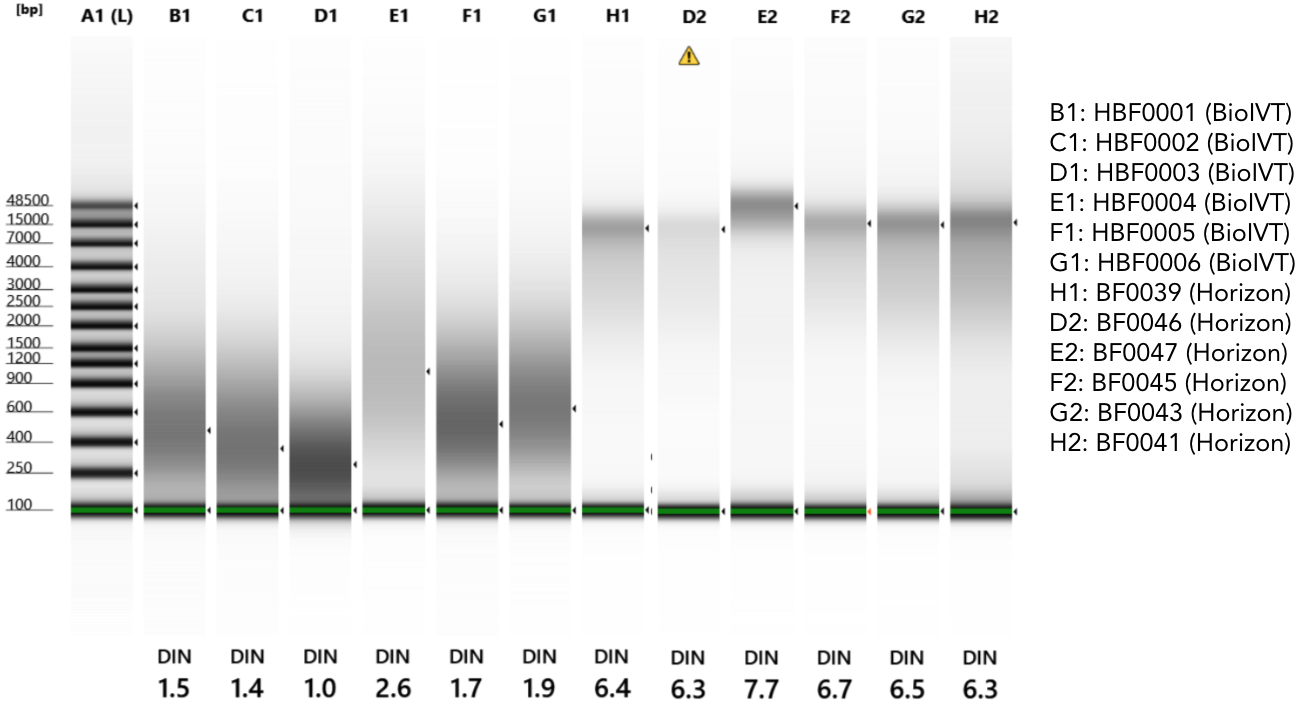


**Supplementary Figure 1**

ScreenTape electrophoresis gel analysis of DNA extracted from FFPE samples. Lane A1:Genomic DNA ladder (Agilent 5067-5366) with associated fragment lengths in base pairs (bp). The FFPE tissue samples (B1-G1) have lower DNA integrity number (DIN) value and shorter fragment size compared to FFPE cell lines (H1; D2-H2). Sample BF0046 (D2) has low DNA concentration (<10ng/µl), outside the working range of the ScreenTape assay (10-100ng/µl).

**Supplementary Figure 2**


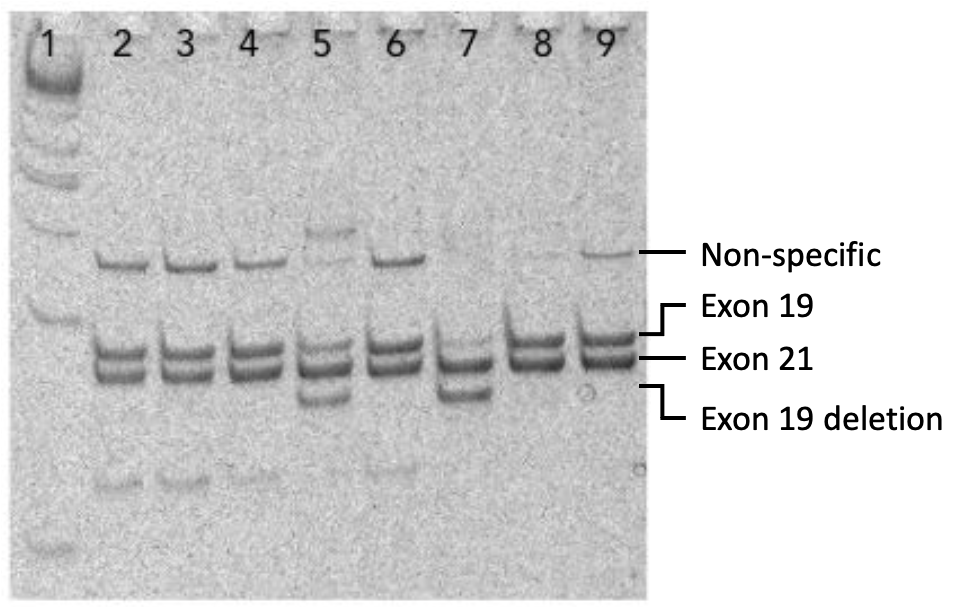


Polyacrylamide gel electrophoresis of multiplex PCRs that amplify *EGFR* exon 19 (92 bp) and exon 21 (84 bp). Lane 1: 50 bp ladder (New England Biolabs B7025). Lane 2; fragmented genomic DNA. Lane 3: HD850 (expected 77 bp band from 1% VAF *EGFR* exon 19 p.E746_A750del). Lane 4: HBF006. Lane 5: HBF005 (expected 77 bp band from *EGFR* exon 19 p.L747_T751del). Lane 6: HBF004. Lane 7: HBF003 (expected 77 bp band from *EGFR* exon 19 p.E746_A750del). Land 8: HBF002. Lane 9: HBF001.


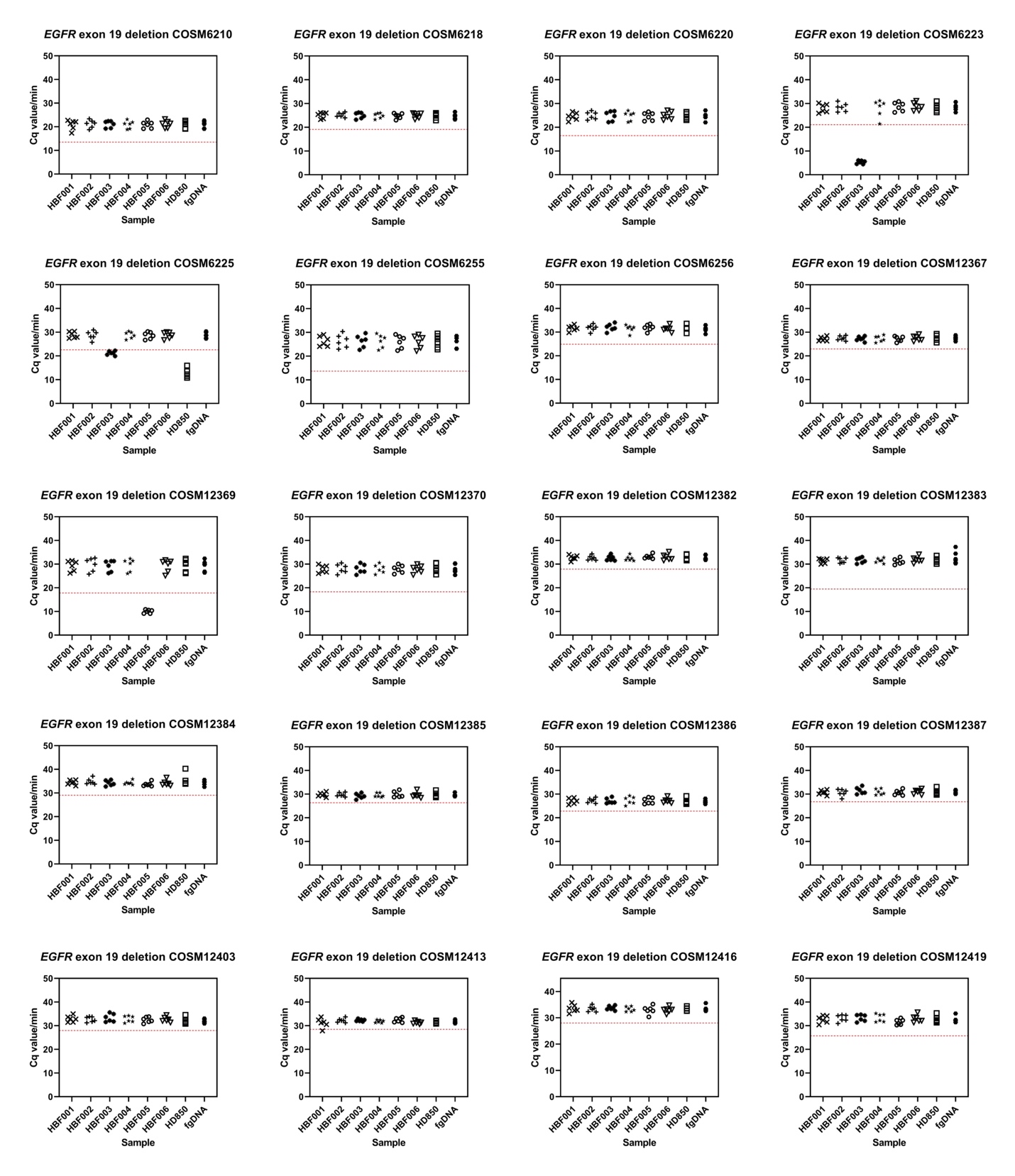


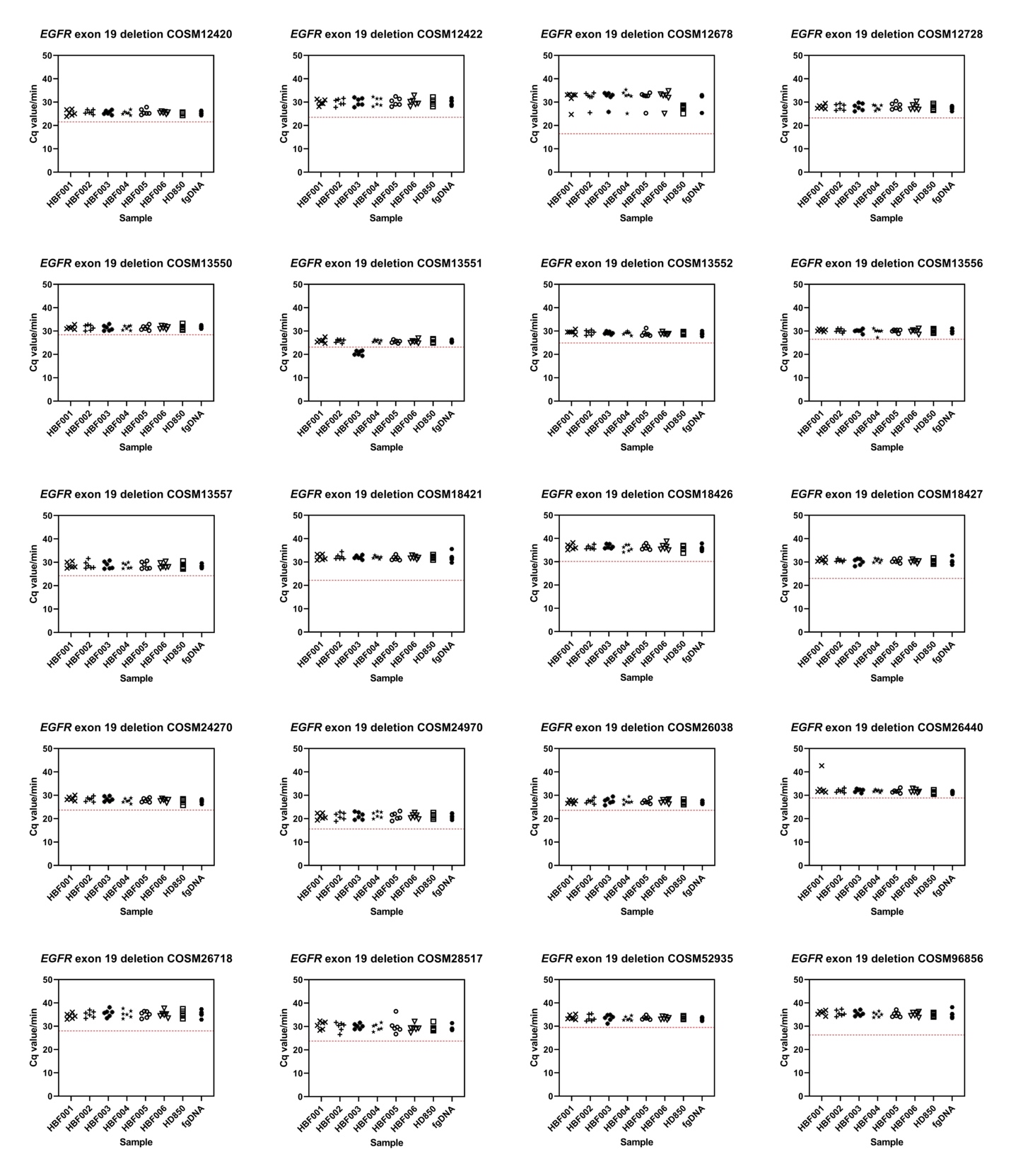


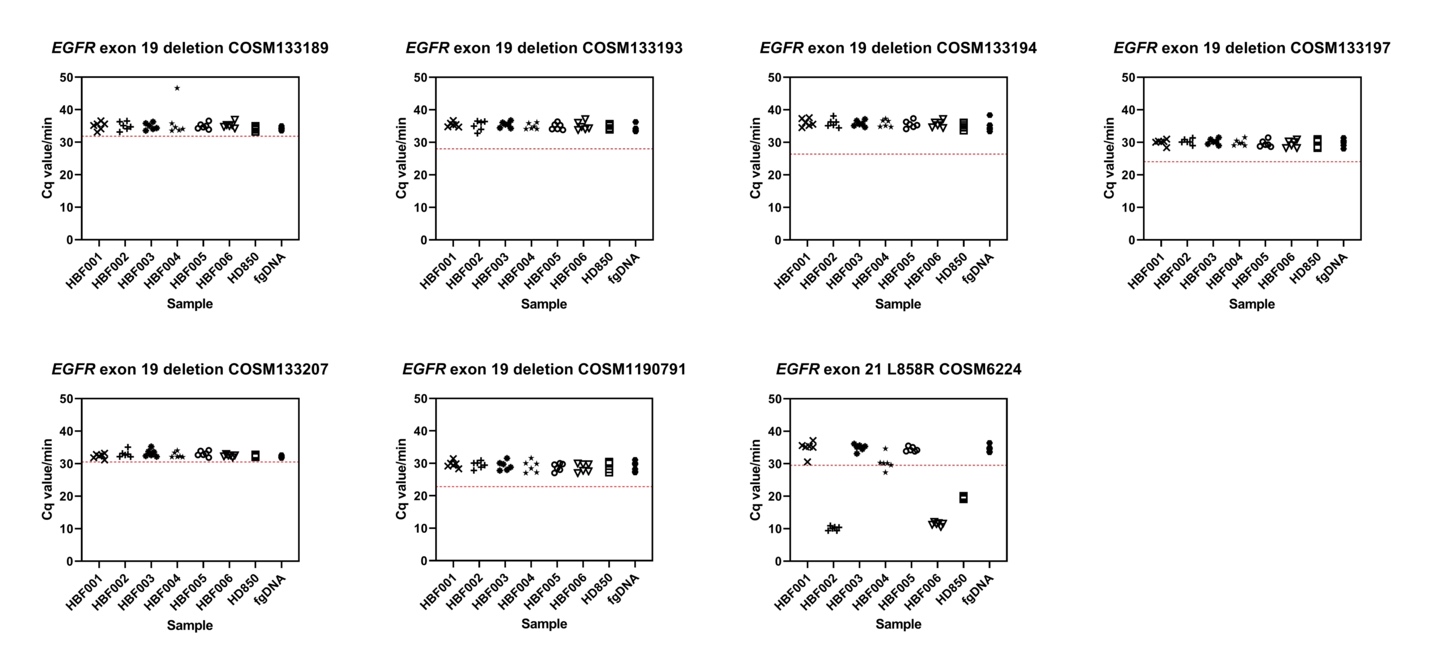


**Supplementary Figure 3**

Analysis of 47 EGFR mutations in FFPE samples from six patients, one positive control (HD850 with known L858R and COSM6225 variants), and one negative control (ultrasonicated DNA, fgDNA). Each sample was run in six independent repeats to demonstrate the reproducibility of ASPYRE to detect mutations in DNA from FFPE samples. The Cq for each repeat is represented by one point on the graph, with different samples represented by a different shape. Samples HBF0001 and HBF0004 are negative for all 47 mutations. HBF0002 and HBF0006 are positive for L858R but negative for exon 19 deletions. Sample HBF0003 was found to contain COSM6223 at high VAF. The similarity of COSM6223 to the sequence of other deletions results in some cross-reactivity and a below-threshold result for two other deletions (COSM6225 & COSM13551), however, the very low Cq observed for the COSM6223 mutation relative to the other two deletions enables this mutation to be clearly identified. Sample HBF0005 is positive for one exon 19 deletion, COSM12369. The Cq threshold for each mutation is shown as a dashed red line. Results below the red dashed line indicate detection of the variant.

**Supplementary Table 1**

Mutation status in FFPE tissue from patients sourced from BioIVT.

| **Identifier** | **Mutation status** |
| --- | --- |
| HBF0001 | Negative |
| HBF0002 | L858R |
| HBF0003 | Exon 19 del |
| HBF0004 | Negative |
| HBF0005 | Exon 19 del |
| HBF0006 | Exon 19 del, L858R |
